# Method of Making Sections of Teeth and Preserving the Delicate Parts

**Published:** 1889-04

**Authors:** Z. A. Latham


### Method of Making Sections of Teeth and Preserving the Delicate Parts.

Z. A. LATHAM.

Take a fresh, or nearly fresh tooth, break in half or cut with a fret saw below the neck, allowing water to drip over it the while, then place in a concentrated solution of mercuric chloride for an hour or so to fix the soft parts; wash in running water for about an hour; then place in thirty per cent. alcohol. After twelve hours place in fifty per cent. of gin; for twelve hours in seventy per cent. To remove the black precipitate, place for twelve hours in ninety per cent. of spirit, to which 1.5—2.0 per cent. tincture of iodine has been added. Then remove the iodine by immersion in absolute alcohol. Keep changing until the teeth become white.

*To Strain.*—Use either alcohol or aqueous solutions of borax carmine. Remove specimens from alcohol, wash in water, often changed or running water, 15-30 minutes then placed in one stain. The aqueous fluid requires one-half days; the alcoholic two-third days. When stained, place in aciduated alcohol [seventy per cent., spirits 100 c. cm.; Hel. (acid muriatic,) 1.0 per cent.,] in which they remain twelve hours; if aqueous solution has been used, 24.36 per cent.; if alcoholic, immerse for fifteen minutes in ninety per cent. spirit. Double this time in absolute alcohol, and finally into an etherial oil for twenty-four hours or more. On removing the specimen from the oil, wash well and quickly with pure xylot, and then place in chloroform for twenty-four hours; then in a solution of chloroform and hard C. balsam for twenty-four hours. To this solution add as much hard balsam as will cover it, into a porcelain dish. Place the dish on a water bath (the teeth left in) and heat gradually to ninety degrees C.; keep at this temperature until a sample of the balsam becomes as hard and like glass upon cooling and being punctured. The teeth are carefully picked out and placed in a vise; thin discs are cut from them with a fret saw, water being allowed to trickle over them the while, and then grind and polish them as usual and mount in chloroform balsam. This may also be used for bone sections.—*From Zeitsch, f. Wiss Micron, v.* 1888, *p.* 300.

